# Association of the DNA Methyltransferase and Folate Cycle Enzymes’ Gene Polymorphisms with Coronary Restenosis

**DOI:** 10.3390/life12020245

**Published:** 2022-02-07

**Authors:** Kalima B. Timizheva, Abdulbary A. M. Ahmed, Amira Ait Aissa, Anna V. Aghajanyan, Leyla V. Tskhovrebova, Madina M. Azova

**Affiliations:** 1 Institute of Medicine, Peoples’ Friendship University of Russia (RUDN University), 117198 Moscow, Russia; ahmed-abdulbari@rudn.ru (A.A.M.A.); ait-aissa-a@rudn.ru (A.A.A.); agadzhanyan-av@rudn.ru (A.V.A.); tskhovrebova-lv@rudn.ru (L.V.T.); azova-mm@rudn.ru (M.M.A.); 2 National Medical Research Center of Cardiology of the Ministry of Healthcare of the Russian Federation, 121552 Moscow, Russia; 3 Federal Research and Clinical Center of Physical-Chemical Medicine of Federal Medical Biological Agency, 119435 Moscow, Russia

**Keywords:** percutaneous coronary intervention, in-stent restenosis, gene polymorphisms, DNA methyltransferase, folate cycle

## Abstract

Background: In recent years, the interest in genetic predisposition studies for coronary artery disease and restenosis has increased. Studies show that polymorphisms of genes encoding folate cycle and homocysteine metabolism enzymes significantly contribute to atherogenesis and endothelial dysfunction. The purpose of this study was to examine some SNPs of genes coding for folate cycle enzymes and DNA methyltransferases as risk factors for in-stent restenosis. Methods: The study included 113 patients after stent implantation and 62 patients without signs of coronary artery disease at coronary angiography as the control group. Real-time PCR and RFLP-PCR were applied to genotype all participants for MTHFR rs1801133, MTHFR rs1801131, MTR rs1805087, MTRR rs1801394, DNMT1 rs8101626, DNMT3B rs1569686, and DNMT3B rs2424913 gene polymorphisms. Statistical data processing was carried out using the R language and the SPSS Statistics 20 software. Results: Statistically significant differences in the DNMT3B gene polymorphisms were found between patients with and without in-stent restenosis. An association of TT rs1569686 and TT rs2424913 genotypes with the development of restenosis was revealed. The TT rs1569686 genotype was more frequent in the patients under the age of 65 years and in the subgroup of patients with post-12-month restenosis, as was the minor GG genotype for MTR rs1805087. The homozygous TT genotype for MTHFR rs1801133 was significantly more frequent in the subgroup over 65 years old. The frequencies of the heterozygous genotype for the MTRR gene and the minor GG homozygotes for the DNMT1 gene were significantly higher in the subgroup with in-stent restenosis under 65 years old. Conclusions: The results of this study could be used for a comprehensive risk assessment of ISR development, determining the optimal tactics and an individual approach in the treatment of patients with coronary artery disease before or after percutaneous coronary interventions, including homocysteine-lowering treatment in patients with hyperhomocysteinemia and a high risk of in-stent restenosis.

## 1. Introduction

Cardiovascular diseases, including coronary artery disease (CAD), are responsible for more than half of all cases of mortality and morbidity worldwide. Percutaneous coronary intervention (PCI) is an invasive procedure that relieves the coronary artery stenosis or occlusion and intensifies the blood supply to the ischemic tissues. The most common method of revascularization is balloon angioplasty of the coronary artery stenotic segments with stent deployment to prevent the coronary artery recoil. The safety and effectiveness of percutaneous coronary revascularization methods have contributed to their widespread adoption and use. Despite the constant technical improvements, the main limiting factor of these methods remains the re-narrowing of the stented artery segment or in-stent restenosis (ISR), the frequency of which is 4–10% in the long-term period [1]. In-stent restenosis is the progressive re-narrowing of the coronary artery stented segment that arises mostly between 3 and 12 months after balloon angioplasty and stent deployment. It generally presents as a recurrence of angina pectoris, but can manifest as acute myocardial infarction in approximately 10% of patients. Introduction of the drug-eluting stent (DES) technology with antiproliferative and antirestenotic effects has significantly improved the short-term outcomes in patients with CAD through improvements in stent design, drugs and polymers [2]. However, in the long-term period, repeated stenosis of the stented artery segment is observed in around 10% of cases, due to the anti-inflammatory and antiproliferative function loss [3,4]. The widespread use of interventional revascularization methods and the sharp increase in the total number of PCI have led to a rise in the global incidence of ISR.

The most frequently described risk factors for ISR development include clinical (gender, age, diabetes mellitus, arterial hypertension, multivessel disease, unstable angina pectoris), angiographic (initial minimum lumen, initial occlusion, stenosis length, proper vessel diameter, bifurcation lesion, ostial lesion, calcification, final minimum lumen) and procedural (total stent length, final balloon size, final pressure, balloon/artery ratio, stenting during dissection) factors [5]. However, these predisposing factors cannot explain all cases of restenosis development after coronary intervention, especially the recurrent narrowing after subsequent stenting in the same patients. In recent decades, a number of studies aimed at finding the pathogenetic mechanisms that underlie restenosis development, as well as determining the complication predictors, have been carried out for modifying the interventional treatment of patients with CAD.

The ISR pathway is based on neointimal hyperplasia resulting from increased smooth muscle cell proliferation and extracellular matrix remodeling. Cytokines and growth factors are activators of these processes; they are released in the non-specific inflammatory response to the presence of the foreign element as the metal struts of the stent in the vessel wall. On average, a sequential cascade of reactions ultimately leads to smooth muscle cell proliferation and migration, narrowing the vessel lumen of the stented artery in 6–9 months after PCI with DES [6,7]. Non-physiological intima repair after stenting can be promoted by the patient’s chronic inflammatory status, which is usually accompanied by endothelial dysfunction.

Homocysteine is a predictor of endothelial dysfunction and may increase platelet aggregation and stimulate smooth muscle cells’ hyperplasia. This effect is achieved through several different mechanisms, such as overactivation of N-methyl-d-aspartate receptors, activation of toll-like receptor 4, impaired Ca2 + processing and increased NADP oxidase activity. An increase in the production of reactive oxygen species and a violation of the synthesis of nitric oxide and reactive oxygen species are observed. The increased production of reactive species during hyperhomocysteinemia is associated with increased expression of several pro-inflammatory cytokines, including interleukins, tumor necrosis factor-alpha and monocyte chemoattractant protein 1 [8,9]. These pathological processes lead to the formation of oxidative stress and endothelial dysfunction, which in turn promotes the development of atherosclerosis and associated cardiovascular diseases.

The role of elevated plasma homocysteine levels as an independent risk factor for the development of atherosclerosis in case of restenosis after PCI has been shown. A meta-analysis of 19 studies revealed a significant increase in the risk of restenosis in the long-term period in patients with elevated plasma homocysteine levels [10,11]. Homocysteine-lowering therapy results in a 40% reduction in the incidence of clinical restenosis. Methylenetetrahydrofolate reductase (MTHFR), 5-methyltetrahydrofolate-homocysteine methyltransferase reductase (MTRR) and 5-methyltetrahydrofolate-homocysteine methyltransferase (MTR) are the folate cycle enzymes responsible for the regulation of homocysteine metabolism [12]. Additionally, hyperhomocysteinemia is accompanied by a decrease in the level of S-adenosylmethionine, the main coenzyme and methyl donor needed for DNA methylation catalyzed by DNA methyltransferases (DNMTs) [13,14]. An experimental model has shown the association of low levels of S-adenosylmethionine with neointimal hyperplasia after balloon angioplasty [15]. On the other hand, it has been established that DNA hypermethylation can contribute to an imbalance of lipoproteins, the development of atherosclerosis and endothelial dysfunction [16].

The aim of this study was to investigate the role of MTHFR rs1801133, MTHFR rs1801131, MTR rs1805087, MTRR rs1801394, DNMT1 rs8101626, DNMT3B rs1569686 and DNMT3B rs2424913 gene polymorphisms as a predisposing factor for in-stent restenosis development in patients with CAD after coronary revascularization.

## 2. Materials and Methods

After coronary angiogram analysis, 175 patients (56.0 ± 10.7 years) were included in the study: 62 patients without signs of CAD were assigned to the control group; 59 patients after balloon angioplasty and stent deployment without in-stent restenosis were assigned to the ISR “-” group; 54 patients with restenosis were assigned to the ISR “+” group. The patients with ISR were classified into subgroups by the terms of the restenosis development (before 12 months, *n* = 22; after 12 months, *n* = 32) and age (under 65 years, *n* = 36; over 65 years, *n* = 18). All subjects gave their informed consent for inclusion before their participation in the study. The study was conducted in accordance with the Declaration of Helsinki, and the protocol was approved by the Local Ethics Committee of the Institute of Medicine of RUDN University (N1, 20 September 2018).

Detailed analysis involved the investigation of clinical symptoms, major risk factors for coronary artery disease (high blood pressure, high low-density lipoprotein cholesterol, low high-density lipoprotein cholesterol, diabetes mellitus, obesity, tobacco smoking and age) and stent characteristics. Repeated angiography was performed one year after PCI or earlier, in the case of symptoms of angina pectoris recurrence or positive tests according to non-invasive methods for myocardial ischemia detection. Clinical restenosis was defined by symptoms or signs of ischemia (ECG assessment or cardiac stress test) and confirmed by the presence of angiographic stenosis in the stented arteries. Diagnosis of ISR was documented in cases with stenosis >50% of a previously stented segment. Subsequent coronaroangiography allowed us to analyze the angiographic risk factors, such as minimal lumen diameter before and after coronary intervention and lesion length. All patients were followed up for at least 24 months. 

Inclusion criteria for the study: over 45 years old, Russians, confirmed atherosclerosis of the coronary arteries or intact arteries according to coronaroangiography. Exclusion criteria: unstable forms of coronary artery disease, decompensated heart failure, renal and hepatic failure, oncological diseases. The primary clinical endpoint of the study was angiographic ISR (stenosis of more than 50% of the stented coronary artery segment). Secondary endpoints of the study were cardiac and non-cardiac death, stent thrombosis, target vessel-related myocardial infarction and clinically driven target lesion revascularization during follow-up period.

We studied polymorphic loci of genes encoding folate cycle enzymes and DNA methyltransferases (Table 1). Genomic DNA was extracted from peripheral blood. 

Genotyping for MTHFR (rs1801133 and rs1801131), MTR (rs1805087) and MTRR (rs1801394) was performed using commercially available kits (Syntol, Russia) for the real-time PCR method. Restriction Fragment Length Polymorphism–Polymerase Chain Reaction (RFLP-PCR) was used for DNMT3B (rs2424913, rs1569686) and DNMT1 (rs8101626) polymorphic loci (Table 2). Detection of restriction products was performed by horizontal electrophoresis on 3% agarose gel.

Statistical analysis of the data was carried out using R language and the SPSS version 20 statistical software. The Chi-square test and Fisher’s exact test were used to compare the frequencies of alleles and genotypes. *p* values less than 0.05 were considered statistically significant. Hardy–Weinberg equilibrium was evaluated by the χ2 test. The odds ratio (OR) and 95% confidence interval (CI) were calculated.

## 3. Results

We performed analysis of clinical, angiographic and genetic factors predisposing patients to the development of CAD and ISR (Table 3 and Table 4). Significant differences were found for diabetes mellitus and multifocal atherosclerosis. It is well known that traditional risk factors for cardiovascular diseases are tobacco smoking and age. The groups of patients with and without ISR were of comparable age (60.0 ± 10.1 and 58.8 ± 8.0, respectively) and frequency of smokers (60% and 58%, respectively). 

Genotype frequencies in the control group for the studied gene polymorphisms were in agreement with the Hardy–Weinberg equilibrium (*p* > 0.05).

Genotype distributions for the MTHFR, MTRR and MTR gene polymorphisms in the group with ISR corresponded to those in the group without restenosis (Table 5). However, stratification of patients with restenosis according to the age revealed significant differences between the subgroups of patients with restenosis who were younger than 65 years old and older. It was found that the homozygous TT genotype for MTHFR rs1801133 was more than three times more frequent in the subgroup over 65 years old (*p* = 0.004), whereas the incidence of AG heterozygotes for MTRR rs1801394 was significantly higher in the subgroup with restenosis in patients under 65 years old (*p* = 0.003).

When analyzing the occurrence of polymorphic variants of the DNMT3B gene, we found that the frequencies of minor TT genotypes for rs1569686 and rs2424913 were significantly higher in the group of patients with ISR (*p* < 0.0001 and *p* = 0.007, respectively) compared with the group of patients without restenosis (Table 5). The rs1569686 TT genotype was also significantly prevalent in patients under 65 years old (*p* = 0.001) and in the subgroup of patients with the development of restenosis after 12 months (*p* = 0.004); GT heterozygosity was more frequent in the group of patients with CAD in comparison with the control group (*p* = 0.03).

Occurrence of the minor homozygous GG genotype for DNMT1 rs8101626 was higher in the subgroup of patients under 65 years old (*p* = 0.04).

## 4. Discussion

S-adenosylmethionine, a universal donor of methyl groups, is formed both from methionine and the ATP molecule, and homocysteine under the control of the MTHFR enzyme [17]. Methionine synthase (MTR) catalyzes homocysteine remethylation. MTR reduction requires methylation catalyzed by the enzyme methionine synthase reductase (MTRR) [18].

The rs1801133 polymorphism of the MTHFR gene encoding methylenetetrahydrofolate reductase is associated with reduced activity of this enzyme, and, as a consequence, with the development of hyperhomocysteinemia [19] and a decrease in the S-adenosylmethionine level. Clinical studies have shown the association of this polymorphism with hyperhomocysteinemia, thrombosis and atherosclerosis [20,21,22]. The TT homozygosity leads to a decrease in the enzyme function by 70% and in heterozygosity by 35%. Some authors found no association of this polymorphism with the development of restenosis after balloon angioplasty of the coronary arteries [23]; however, further studies of its relationship with ISR were not carried out. We found that TT homozygosity is an ISR risk factor for patients over 65 years old (CI 1.255–7.173; OR 3.0).

The MTHFR rs1801131 polymorphism is not associated with angiographically confirmed atherosclerosis in the Chinese population [24]. Our results also showed the absence of a significant association of the polymorphism with CAD and restenosis in general; however, the stratification of patients by the terms of ISR development revealed a possible role of the homozygous CC genotype in the development of early restenosis (CI 1.028–5.938; OR 2.470).

The polymorphism of the MTR rs1805087 gene (AG and GG genotypes), resulting in a decrease in the activity of the enzyme methionine synthase, is associated with the development of atherosclerosis [25]. In our study, the heterozygous AG genotype was more prevalent in the group of patients with coronary artery disease; however, the difference did not reach a significant level. We found that the homozygous GG genotype predisposed individuals to late restenosis (CI 1.170–16.043; OR 4.333).

Polymorphic AG and GG variants for MTRR rs1801394 are associated with changes in the methionine synthase reductase conformation and a decrease in its activity, leading to impaired folate metabolism, hyperhomocysteinemia and atherosclerosis [26,27]. According to our findings, the AG heterozygotes were more frequent among patients under 65 years old, and GG homozygotes were more frequent in the group with late restenosis. 

The transfer of methyl groups to cytosine DNA nucleotides is catalyzed by a group of DNA methyltransferases (DNMT1, DNMT3A, DNMT3B), in which S-adenosylmethionine is used as a methyl group donor and thereafter converted into homocysteine [28,29]. The role of DNMT1 rs8101626, DNMT3B rs1569686 and DNMT3B rs2424913 in the development of various oncological diseases and pathology of pregnancy has been revealed [16,30,31]. Although rs2424913 CT/TT genotypes are associated with hypermethylation, the influence of other gene polymorphisms on DNA methylation is controversial. It has been shown experimentally that pharmacological inhibition of DNA hypermethylation leads to a decrease in the SMCs’ proliferation and neointima formation [32,33]. As far as we know, the presented work is the first to assess the role of the above-described polymorphisms in the development of CAD and ISR. We found that the TT rs1569686 genotype is associated with the development of ISR in general (CI 2.658–16.778; OR 6.679), while rs2424913 TT has a tendency to be associated with it (CI 0.689–6.666; OR 2.143). The rs8101626 GG may be considered as a marker for protection against ISR in patients older than 65 years (CI 0.096–0.883; OR 0.291), while the rs1569686 TT genotype leads to a predisposition to ISR in patients under 65 years of age (CI 3.240–19.426; OR 7.933) and late restenosis (CI 3.143–18.756; OR 7.677).

## 5. Conclusions

Our findings prove the notion of the necessity of patients’ stratification by age and term of restenosis development to reveal the genetic factors predisposing them to this complication. Thus, TT genotypes for DNMT3B rs1569686 and DNMT3B rs2424913 are associated with ISR in general. The MTHFR rs1801133 TT genotype is a specific risk factor for patients over 65 years old, whereas DNMT1 rs8101626 GG acts as a marker for protection for such patients. Younger individuals are predisposed to restenosis if they have DNMT3B rs1569686 TT or MTRR rs1801394 AG genotypes. Early restenosis is associated with homozygosity for the MTHFR rs1801131 CC genotype; late restenosis is more frequent in MTR rs1805087 GG and DNMT3B rs1569686 TT homozygotes. The results of this study can be used for a comprehensive risk assessment of ISR development, determining the optimal tactics and an individual approach in the treatment of patients with coronary artery disease before or after percutaneous coronary interventions, including homocysteine-lowering treatment in patients with hyperhomocysteinemia and a high risk of ISR.

## Figures and Tables

**Table 1:** Studied genes and polymorphisms.

Gene (Symbols, Title)	OMIM Number	Location	Reference Transcript	SNP	Ref SNP ID
MTHFR 5,10-Methylenetetrahydrofolate reductase	607093	1p36.22	NM_005957.5	C677T	rs1801133
				A1298C	rs1801131
MTR 5-methyltetrahydrofolate-homocysteine S-methyltransferase	156570	1q43	NM_000254.2	A2756G	rs1805087
MTRR methionine synthase reductase (MSR)	602568,	5p15.31	NM_002454.2	Ile22Met	rs1801394
DNMT1 DNA methyltransferase 1 (DNMT, MCMT, CXXC9)	126375	19p13.2	NM_001379.2	A626G	rs8101626
DNMT3B DNA methyltransferase 3 beta	602900	20q11.21	NM_006892.3	C149T	rs2424913
				G39179T	rs1569686

**Table 2:** Genotyping conditions.

Gene Polymorphism	Primer Sequences	Annealing Temperature	Restriction Enzymes	DNA Fragments, bp
DNMT3B rs2424913	F: 5′TGCTGTGACAGGCAGAGCAG3′ R: 5′GGTAGCCGGGAACTCCACGG3′	65 °C	ASPA2I	CC: 380 CT:380, 207, 173 TT: 207, 173
DNMT3B rs1569686	F: 5GAGGTCTCATTATGCCTAGG3′ R: 5GGGAGCTCACCTTCTAGAAA3′	49 °C	PVuII	TT: 132, 93 TG: 225, 132, 93 GG: 225
DNMT1 rs8101626	F: 5′-CAAATGGGCCACCTAGACAC-3′ R: 5′-GGCAGAGATTGAGCCAGAAG-3′	67 °C	BStMAI	AA: 640 AG: 640, 474, 166 GG: 474, 166

**Table 3:** Stent characteristics in studied groups of patients.

Characteristics	With ISR	Without ISR
Total length of stents, mm (M ± SD)	52.5 ± 40.2	47.0 ± 29.0
Minimal stent diameter, mm (M ± SD)	3.1 ± 0.54	2.9 ± 0.45
I generation DES, %	20	17
II generation DES, %	66	62
III generation DES, %	14	21

**Table 4:** General characteristics of studied groups of patients.

	With ISR (*n* = 54)	Without ISR (*n* = 59)	*p* Value	Control (*n* = 62)
Age, years (M ± SD)	60.0 ± 10.1	58.8 ± 8.0	0.885	49.7 ± 10.8
Smoking, %	60	58	0.774	28
Obesity, %	34	27	0.282	21
Dyslipidemia, %	62	52	0.153	14
Diabetes mellitus, %	53 *	8	<0.0001	0
Myocardial infarction, %	36	25	0.091	0
Multifocal atherosclerosis, %	47 *	20	0.0001	0
Arterial hypertension, %	63	56	0.313	52
Systolic blood pressure, mm Hg (M ± SD)	138 ± 7.3	137 ± 7.8	0.952	129 ± 11.2
Diastolic blood pressure, mm Hg (M ± SD)	88 ± 4.9	87 ± 7.8	0.939	79 ± 9.7
Glucose, mmol/L (M ± SD)	4.4 ± 1.0	4.3 ± 1.2	0.973	4.4 ± 0.7
Total cholesterol, mmol/L (M ± SD)	5.2 ± 1.7	4.3 ±1.8	0.770	4.2 ± 0.8
Low-density lipoproteins, mmol/L (M ± SD)	2.3 ± 1.2	2.4 ± 0.9	0.963	2.4 ± 0.5
High-density lipoproteins, mmol/L (M ± SD)	1.4 ± 0.6	1.5 ± 0.5	0.953	1.6 ± 0.5
Multivessel coronary artery disease, %	68 *	47	0.004	0

Note: *—significantly different from the group of patients without restenosis (*p* < 0.05).

**Table 5:** Frequencies of genes and genotypes (%) in the studied groups.

Gene Polymorphisms	Genotypes and Alleles	Control (*n* = 62)	CAD (*n* = 113)	Restenosis+ (*n* = 54)	Restenosis– (*n* = 59)	Restenosis+				*p* Value
						Under 65 Years (*n* = 36)	Over 65 Years (*n* = 18)	Before 12 Months (*n* = 22)	After 12 Months (*n* = 32)	
MTHFR rs 1801133	CC	50% (31)	51% (58)	48% (26)	54% (32)	50% (18)	44% (8)	50% (11)	47% (15)	
	CT	39% (24)	39% (44)	41% (22)	37% (22)	44% (16)	34% (6)	41% (9)	41% (13)	
	TT	11% (7)	10% (11)	11% (6)	9% (5)	6% (2) ^1,^*	22% (4) ^4^	9% (2)	12% (4)	*p* = 0.004 ^1^ *p* = 0.037 ^4^
	C	69.5%	70.5%	68.5%	72.5%	72%	61%	70.5%	67.5%	
	T	30.5%	29.5%	31.5%	27.5%	28%	39%	29.5%	32.5%	
MTHFR rs 1801131	AA	34% (21)	26% (29)	22% (12)	29% (17)	22% (8)	22% (4)	18% (4)	25% (8)	
	AC	58% (36)	60% (68)	65% (35)	56% (33)	64% (23)	67% (12)	59% (13)	69% (22)	
	CC	8% (5)	14% (16)	13% (7)	15% (9)	14% (5)	11% (2)	23% (5) ^2,^*	6% (2)	*p* = 0.003 ^2^
	A	63%	56%	54.5%	57%	54%	55.5%	47.5%	59.5%	
	C	37%	44%	45.5%	43%	46%	44.5%	52.5%	40.5%	
MTR rs 1805087	AA	66% (41)	55% (63)	56% (30)	56% (33)	53% (19)	61% (11)	55% (12)	56% (18)	
	AG	28% (17)	38% (43)	35% (19)	41% (24)	36% (13)	33% (6)	41% (9)	31% (10)	
	GG	6% (4)	7% (7)	9% (5)	3% (2)	11% (4)	6% (1)	4% (1) ^2^	13% (4) ^4^	*p* = 0.046 ^2^ *p* = 0.022 ^4^
	A	80%	74%	73.5%	76.5%	71%	77.5%	75.5%	71.5%	
	G	20%	26%	26.5%	23.5%	29%	22.5%	24.5%	28.5%	
MTRR rs 1801394	AA	23% (14)	22% (25)	20% (11)	24% (14)	14% (5)	33% (6)	27% (6)	16% (5)	
	AG	42% (26)	43% (49)	46% (25)	41% (24)	53% (19) ^1,^*	34% (6)	50% (11)	43% (14)	*p* = 0.003 ^1^
	GG	35% (22)	35% (39)	34% (18)	35% (21)	33% (12)	33% (6)	23% (5) ^2,^*	41% (13)	*p* = 0.015 ^2^
	A	44%	43.5%	43%	44.5%	40.5%	50%	52%	37.5%	
	G	56%	56.5%	57%	55.5%	59.5%	50%	48%	62.5%	
DNMT1 rs8101626	AA	53% (33)	35% (39)	35% (19)	34% (20)	34% (12)	39% (7)	41% (9)	31% (10)	
	AG	37% (23)	51% (58)	52% (28)	51% (30)	50% (18)	56% (10)	45% (10)	56% (18)	
	GG	10% (6)	14% (16)	13% (7)	15% (9)	16% (6) ^1^	5% (1)	14% (3)	13% (4)	*p* = 0.04 ^1^
	A	71.5%	60.5%	61%	59.5%	59%	67%	63.5%	59%	
	G	28.5%	39.5%	39%	40.5%	41%	33%	36.5%	41%	
DNMT3B Rs1569686	GG	56% (35)	49% (55)	28% (15)	68% (40)	30% (11)	22% (4)	22% (5)	31% (10)	
	GT	20% (12)	36% (41) ^3,^*^,5,^*	50% (27)	24% (14)	42% (15)	67% (12)	64% (14)	41% (13)	*p* = 0.01 ^3^ *p* = 0.03 ^5^
	TT	24% (15)	15% (17)	22% (12) ^4,^*	8% (5)	28% (10) ^1,^*	11% (2)	14% (3) ^2,^*	28% (9)	*p* = 0.001 ^1^ *p* = 0.004 ^2^ *p* < 0.0001 ^4^
	G	66%	67%	53%	80%	51%	55.5%	54%	51.5%	
	T	34%	33%	47%	20%	49%	44.5%	46%	48.5%	
DNMT3B Rs2424913	CC	40% (25)	40% (45)	35% (19)	25% (26)	30% (11)	44% (8)	32% (7)	38% (12)	
	CT	47% (29)	50% (56)	50% (27)	70% (29)	53% (19)	44% (8)	54% (12)	47% (15)	
	TT	13% (8)	10% (12)	15% (8) ^4,^*	5% (4)	17% (6)	12% (2)	14% (3)	15% (5)	*p* = 0.007 ^4^
	C	63.5%	65%	60%	60%	56.5%	66%	59%	61.5%	
	T	36.5%	35%	40%	40%	43.5%	34%	41%	38.5%	

Note: ^1^—significantly different from the group of patients with restenosis over 65 years, ^2^—significantly different from group of patients with restenosis after 12 months, ^3^—significantly different from group of patients with restenosis, ^4^—significantly different from the group of patients without restenosis, ^5^—significantly different from the control group (*p* < 0.05), *—significant after Bonferroni correction.

## Data Availability

The data that support the findings of this study are not publicly available due to patient confidentiality.
